# Supplementary figures and images for: Real-time PCR assay for detection and differentiation of Coccidioides immitis and Coccidioides posadasii from culture and clinical specimens
Source: PLoS Negl Trop Dis. 2021 Sep 16;15(9):e0009765. doi: 10.1371/journal.pntd.0009765 (PMC8486383; doi:10.1371/journal.pntd.0009765)

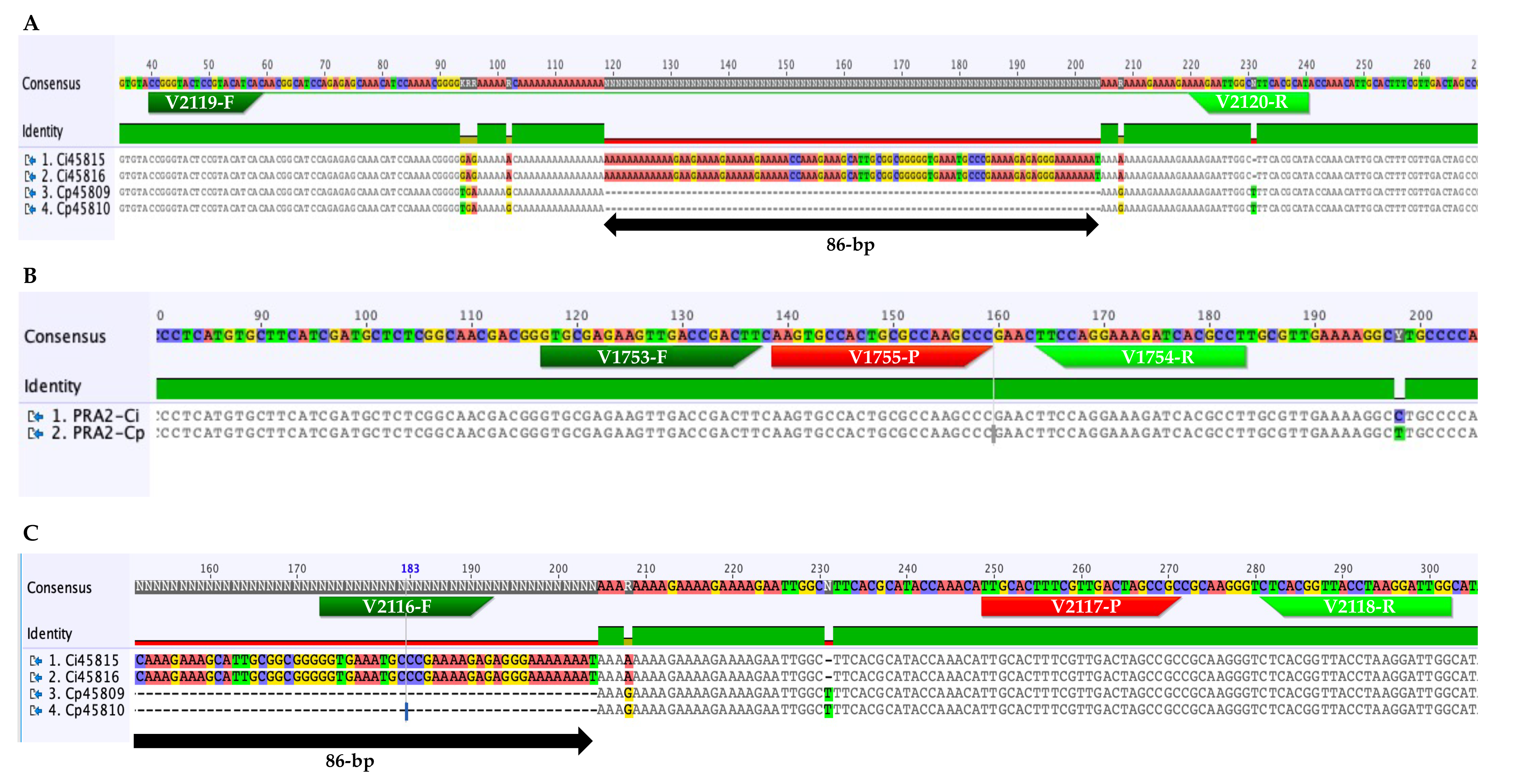

Supplement: S1 Fig — (A) Nucleotide sequences of PCR amplicons of C. immitis (Ci45815 & Ci45816) and C. posadasii (Cp45809 & Cp45810) contigs [17] were aligned using Geneious R9 9.1.6 software (Biomatters, Inc., San Diego, CA). The gray background defines the consensus sequences between C. immitis and C. posadasii while non-consensus sequences are highlighted in color. Conventional PCR forward (V2119-F) and reverse (V2020-R) primers were designed from the consensus region flanking the deleted region (86-bp) of C. posadasii. As a result, primer set produced 114-bp product for C. posadasii and 200-bp product for C. immitis. (B) PRA2 gene from both C. immitis and C. posadasii were aligned and consensus region of the gene was used for the design of forward (V1753-F), and reverse (V1754-R) primers, and probe (V1755). The resulting primers and probe produced amplicons against both C. immitis and C. posadasii. (C) C. immitis (Ci45815 & Ci45816) and C. posadasii (Cp45809 & Cp45810) contigs were aligned as described in S1A Fig. The forward primer (V2116-F) was designed from C. immits contig region deleted in C. posadasii while reverse primer (V2118-R) and probe (V2117) were designed from consensus region of the contigs. The resulting primers and probe produced amplicon against C. immits, but not against C. posadasii. (TIF) [file pntd.0009765.s001.tif]
