# Supplementary material for: Real-time PCR assay for detection and differentiation of Coccidioides immitis and Coccidioides posadasii from culture and clinical specimens
Source: PLoS Negl Trop Dis. 2021 Sep 16;15(9):e0009765. doi: 10.1371/journal.pntd.0009765 (PMC8486383; doi:10.1371/journal.pntd.0009765)
Supplement: Attachment — Submitted filename: Response to reviewers comments_Sc081421.docx [file pntd.0009765.s008.docx]

**Response to Reviewers queries**

We thank all three reviewers for their valuable comments on our manuscript. We made revisions in response to these comments and suggestions. Our responses are provided in bold letters.

Reviewer #1: The objective of the study are clearly articulated. But this study is no a hypothesis-based study. It is a methods-based study. The study design does address the stated objectives.

The samples and populations are clearly described but a hypothesis is not being tested.

**-This is a method development study and therefore, quality validation parameters were tested in lieu of a prior hypothesis.**

The statistical test were not adequately described. On Page 10 in the Statistical analysis section, the authors should state the statistical test performed e.g., t-test, anova etc. including the post-hoc tests.

**- Statistical analysis is now provided on page 9, lines 241-245.**

Yes, the ethical and regulatory requirements seem to be met.

Reviewer #2: While statistical testing was briefly mentioned, it is extremely vague and no specific tests were mentioned. No evidence from statistical tests are given in any of the tables.

- **We apologize for this deficiency. The revised manuscript contains statistical analysis section on page 9, lines 241-245.**

Reviewer #3: Chuterverdi et al have provided a very nice article on the development of RT-PCR testing for Coccidioides spp.  There methods are sound, all relevant information is provided, and their labs has a history of strong work in similar areas.  I do not have any methodologic concerns.

--------------------

<b>Results</b></br></br>

-Does the analysis presented match the analysis plan?</br>

-Are the results clearly and completely presented?</br>

-Are the figures (Tables, Images) of sufficient quality for clarity?</br></br>

Reviewer #1: Yes.

Reviewer #2: (No Response)

Reviewer #3: Results are very easy to follow, are justified by the data, and clear.

--------------------

<b>Conclusions</b></br></br>

-Are the conclusions supported by the data presented?</br>

-Are the limitations of analysis clearly described?</br>

-Do the authors discuss how these data can be helpful to advance our understanding of the topic under study?</br>

-Is public health relevance addressed?</br></br>

Reviewer #1: Yes.

Reviewer #2: (No Response)

Reviewer #3: Conclusions are sound.  Dsicussion should be improved a bit with additional references added, and the clinical usefulness of RT-PCR discussed.  The results of Barker et al with regard to spp specific differences and how this may be of clinical significance (not just epi significance) can be added.

**Thanks for bringing this excellent point. We have expanded introductions and discussion and cited Barker et al 2019 and Kirkland and Fierer 2018.**

--------------------

<b>Editorial and Data Presentation Modifications?</b></br><br/>

Use  this section for editorial suggestions as well as relatively minor modifications of existing data that would enhance clarity. If the only modifications needed are minor and/or editorial, you may wish to recommend “Minor Revision” or “Accept”.

Reviewer #1: On Page 10 in the Statistical analysis section, the authors should state the statistical test performed e.g., t-test, anova etc. including the post-hoc tests.

**-This information is now provided in the revised manuscript.**

Page 11: Lines 1-2 Amplification efficiency is important information to this study because the efficiency can impact the limit of detection. Amplification efficiency will also allow reviewers to access how reliable the assay is. Good quality assay typically have amplification efficiencies of 90 - 110 %. Please, add this data.

**-The amplification efficiency is added to Fig. 2. The assay is extremely sensitive with detection limit of 1 pg gDNA per PCR reaction. The assay is highly specific with no cross reaction seen with both closely and distantly related fungal pathogens to *Coccidioides* species.**

Reviewer #2: The methods can be difficult to follow at times. It would benefit from subheadings, especially in the DNA extraction section, to differentiate between protocols for the various sample types. The manufacturers' info is not always complete in this section and the formatting of units is not consistent.

**-The method section is expanded as per reviewers’ suggestion. We have used Qiagen kit and QIAcube semi-automated extraction platform throughout this investigation for all sample types.**

The PCR sections may benefit from diagrams, particularly for the primer and probe design.

**-We have added Supplementary Figure 1 A, B and C highlighting sequences with the location of the primers and probes.**

Many of the tables contain raw data, rather than summaries for sample types, and are not interpretation-friendly. The manuscript would benefit if the raw data is kept in the supplement and interpretations presented in the main manuscript.

**-We agree with the reviewer’s comments. The summary of all tables is now part of Table 1 and raw data is now part of Supplementary Tables 1 to 6.**

Reviewer #3: Italicize Coccidioides throughout (page 5 – line 3 etc; why were continuous line numbers not used throughout? Far simpler for reviewers).

**-We apologize for missing italicization, it is corrected. Also, the revised manuscript has continuous line numbers.**

Page 15, lines 3-8; this has previously been described by Taylor et al and Barker et al.  Appropriate references could be added.

--------------------

<b>Summary and General Comments</b></br></br>

Use  this section to provide overall comments, discuss strengths/weaknesses of the study, novelty, significance, general execution and scholarship. You may also include additional comments for the author, including concerns about dual publication, research ethics, or publication ethics. If requesting major revision, please articulate the new experiments that are needed.

Reviewer #1: What are the main claims of the paper and how significant are they for the discipline?

Coccidioidomycosis (Valley Fever) is a pulmonary and systemic fungal disease with increasing incidence and expanding endemic areas. The differentiation of two Coccidioides species remains problematic in clinical laboratories as conventional methods are limited by complexity or time constraints. Therefore, the authors developed TaqMan-probes for a duplex qPCR assay for rapid differentiation of C. immitis and C. posadasii from culture and clinical specimens.

Are these claims novel? If not, which published articles weaken the claims of originality of this one?

Although the techniques used here are not novel per se, the TaqMan-probes developed here overcome short comings of current techniques for diagnosis of Coccidioidomycosis.

Are the claims properly placed in the context of the previous literature? Have the authors treated the literature fairly?

The claims appear to be properly placed in the context of previous literature.

Do the data and analyses fully support the claims? If not, what other evidence is required?

The data partially support the claims made here.

On Page 10 in the Statistical analysis section, the authors should state the statistical test performed e.g., t-test, anova etc. including the post-hoc tests.

Would additional work improve the paper? How much better would the paper be if this work were performed and how difficult would it be to do this work?

I don’t believe that additional work is necessary.

PLOS Neglected Tropical Diseases encourages authors to publish detailed protocols and algorithms as supporting information online. Do any particular methods used in the manuscript warrant such treatment? If a protocol is already provided, for example for a randomized controlled trial, are there any important deviations from it? If so, have the authors explained adequately why the deviations occurred?

No.

Is this paper outstanding in its discipline? If yes, what makes it outstanding? If not, why not?

The authors have developed a technique with advantages over current methodologies.

If the paper is considered unsuitable for publication in its present form, does the study itself show sufficient potential that the authors should be encouraged to resubmit a revised version?

No. With very minor revision the paper is suitable for publication.

Are original data deposited in appropriate repositories and accession/version numbers provided for genes, proteins, mutants, diseases, etc.?

N/A

Are details of the methodology sufficient to allow the experiments to be reproduced?

Page 11: Lines 1-2 Amplification efficiency is important information to this study because the efficiency can impact the limit of detection. Please, add this data instead of stating data not shown.

Is the manuscript well organized and written clearly enough to be accessible to non-specialists?

Yes.

Reviewer #2: Financial disclosure section has not been completed to the specifications of the journal.

Reviewer #3: (No Response)

--------------------

PLOS authors have the option to publish the peer review history of their article (<a href="<https://journals.plos.org/plosntds/s/editorial-and-peer-review-process#loc-peer-review-history>" target="_blank">what does this mean?</a>). If published, this will include your full peer review and any attached files.<br><br>

If you choose “no”, your identity will remain anonymous but your review may still be made public.<br><br>

<b>Do you want your identity to be public for this peer review?</b> For information about this choice, including consent withdrawal, please see our <a href="<https://www.plos.org/privacy-policy>" target="_blank">Privacy Policy</a>.

Reviewer #1: No

Reviewer #2: No

Reviewer #3: No

Figure Files:

While revising your submission, please upload your figure files to the Preflight Analysis and Conversion Engine (PACE) digital diagnostic tool, <https://protect2.fireeye.com/v1/url?k=546b2375-0bf01a7b-5469da40-0cc47aa88e08-662de03c7a348402&q=1&e=0f371b1f-e7e6-4807-b7da-28f59cef02bd&u=https%3A%2F%2Fpacev2.apexcovantage.com%2F>. PACE helps ensure that figures meet PLOS requirements. To use PACE, you must first register as a user. Then, login and navigate to the UPLOAD tab, where you will find detailed instructions on how to use the tool. If you encounter any issues or have any questions when using PACE, please email us at [figures@plos.org](mailto:figures@plos.org).

Data Requirements:

Please note that, as a condition of publication, PLOS' data policy requires that you make available all data used to draw the conclusions outlined in your manuscript. Data must be deposited in an appropriate repository, included within the body of the manuscript, or uploaded as supporting information. This includes all numerical values that were used to generate graphs, histograms etc.. For an example see here: <https://protect2.fireeye.com/v1/url?k=2c7f045e-73e43d50-2c7dfd6b-0cc47aa88e08-6983c2f03e080e65&q=1&e=0f371b1f-e7e6-4807-b7da-28f59cef02bd&u=http%3A%2F%2Fwww.plosbiology.org%2Farticle%2Finfo%253Adoi%252F10.1371%252Fjournal.pbio.1001908%23s5>.

Reproducibility:

To enhance the reproducibility of your results, we recommend that you deposit your laboratory protocols in protocols.io, where a protocol can be assigned its own identifier (DOI) such that it can be cited independently in the future. Additionally, PLOS ONE offers an option to publish peer-reviewed clinical study protocols. Read more information on sharing protocols at <https://plos.org/protocols?utm_medium=editorial-email&utm_source=authorletters&utm_campaign=protocols>

__________________________________________________

In compliance with data protection regulations, you may request that we remove your personal registration details at any time.  (Use the following URL: <https://www.editorialmanager.com/pntd/login.asp?a=r>). Please contact the publication office if you have any questions.
